# The Expressive Therapies Continuum as a Migratory Journey: A Classroom Experience Through the Lenses of a Teacher, a Special Educator, and Co-Art Therapists

**DOI:** 10.3390/bs16020285

**Published:** 2026-02-15

**Authors:** Maria Riccardi, Pierre Plante, Tamara Vieira

**Affiliations:** 1 Department of Psychology, Université du Québec, Montréal, QC H2X 3P2, Canada; plante.p@uqam.ca; 2 École d’art-thérapie, Université du Québec en Abitibi-Témiscamingue, Montréal, QC H3A 1K2, Canada; 3 School of Communication Sciences and Disorders, Faculty of Medicine and Health Sciences, McGill University, Montréal, QC H3A 1G1, Canada; tamara.vieira@mcgill.ca

**Keywords:** Expressive Therapies Continuum (ETC), trauma, art therapy, trauma integration

## Abstract

This phenomenologically informed qualitative study gives voice to the experience of a teacher, a special educator, and co-art therapists in art therapy workshops given to first-generation immigrant adolescents in a welcome classroom in Quebec, Canada. This study uses a constructivist–interpretive paradigm, allowing the exploration of individual and interactional dynamics in artmaking. The objective was to explore the experiences and perceptions of a teacher, a special educator, and two art therapists who participated in art-based workshops in a welcome classroom for adolescents, and to understand the meaning these workshops hold for them as well as their perception of the meaning it holds for the young people. Grounded in the Expressive Therapies Continuum (ETC), 17 first-generation immigrant adolescents, their teacher, the special educator, and two art therapists participated in nine art therapy workshops and an art exhibition to foster creativity, openness, and reciprocity. The adolescents had experienced trauma, including wars, violence, and separation, as well as uprooting, and acculturation in the host country. Given the limited research on school-based art therapy workshops in high schools, this study seeks to address that gap by examining how students in a welcome class emerge, unfold, and express themselves through the perspectives of the supporting adults. Reflexive thematic analyses revealed that the art workshops were an emancipatory experience, an existential path to crossing barriers, and a lived space for self-expression. These findings highlight the ETC’s potential in helping immigrant adolescents and their classroom community share their stories and they emphasize art therapy’s transcultural value.

## 1. Introduction

Art therapy is an effective therapeutic modality for newly arrived immigrants or refugees with traumatic experiences (McLeod et al., 2021; Rousseau & Frounfelker, 2019). Contemplation of artwork can lead to a true encounter and an authentic dialog with the world (Gadamer, 1960/1996). In collaborative environments, young adolescents often discover their ability to shape their existence and embrace new ways of interacting. Therefore, integrating art into classroom settings as a shared experience may reduce trauma-related symptoms in students (Beauregard et al., 2017, 2024).

This study is a component of one of two scientific articles that comprise part of a doctoral thesis embedded within larger qualitative research inquiry. The dissertation adopts an Interpretative Phenomenological Analysis (IPA) methodology (Smith et al., 2022), aimed at deepening our understanding of the artistic development of adolescents and the supporting adults in the context of a welcome class (“classe d’accueil”). Welcome classes are specialized classrooms in Quebec that focus on supporting newly arrived students with the acquisition of French language and integration into the school system (Ministère de l’Éducation du Québec, 2024). The qualitative studies based on hermeneutic phenomenology comprised two components: art therapy workshops guided by the Expressive Therapies Continuum (ETC), followed by semi-structured interviews with students, the homeroom teacher, the special educator, and the co-art therapists. Guided by a phenomenological perspective, the analysis of the participants’ experience was supplemented by a reflexive process, supported by the researcher’s logbook (Fish, 2025).

The aim of the first study was to explore the lived experiences of immigrants and displaced youth participating in the arts. Through image-making, the students could map their past experiences, co-create a shared world, and reimagine their migratory process. This present study is phenomenologically informed, adopts a predominantly inductive, data-driven approach in the analysis and presents a reflexive thematic analysis of the interviews conducted with the key adults involved in the art therapy workshops (Braun & Clarke, 2022). The latter fulfilled the sub-objective of gathering a first-person account of the perspectives of the homeroom teacher, the special educator, and the art therapists on their participation in the art-based program, in relation to the experiences of young people. Consequently, both studies explored the same workshops, the first focusing on the point of view of the children, while the second emphasizes the adults’ perceptions. The supporting adults generated a rich account of their experiences as a means of understanding their perception of the workshops, and thus their role in the integration of the arts by the students (Motta & Larkin, 2023). The combined perspectives of the supportive adults allowed for a better understanding of how the ETC offered the students a unique understanding of oneself, others, and the world around them—a discussion initiated in the first study from the point of view of the students.

Art therapy can serve as a valuable support for displaced individuals (Annous et al., 2022; Feen-Calligan et al., 2020; Pirotte et al., 2025) emerging as an effective means of communication (Akthar & Lovell, 2019; Feen-Calligan et al., 2020). A phenomenological lens helps clarify the meaning attributed by school practitioners and students to the creative experience of the arts, which offers them a temporary sense of home throughout the resettlement process (Dieterich-Hartwell & Koch, 2017). Therefore, the phenomenology of images may work with a real presence that allows young people to reconnect with their creativity (Plante et al., 2023). Paradoxically, there is limited research with newcomers that has focused on high school programs and offers an existential perspective based on the ETC framework. Therefore, this study aimed to better understand and described the supporting adults’ experiences and perceptions of the workshops and its influence on the students.

### 1.1. Migratory Loss as a Shared Experience

School is the first place that youth from migratory populations attend in their host countries. In 2024, approximately 123.2 million people were forcibly displaced globally, including refugees, asylum seekers, as well as those internally displaced or needing protection (World Health Organization [WHO], 2025). In this study, ‘migratory populations’ refers to immigrants who have chosen to settle in another country, asylum seekers who have applied for legal protection due to threats to life, and refugees who fear persecution in their home country (Alharfoush, 2025; Statistique Canada, 2025). Moving to a new country often implies the loss of significant cultural, linguistic, social, and familial ties; challenges during the journey; and experiences of grief in the host country (Alharfoush, 2025). Bereavement, and symptoms of anxiety, depression, and post-traumatic stress disorders are particularly prevalent in migrant populations, especially refugees and those living in precarious situations (Giacco et al., 2017; Hamouda et al., 2024; Rousseau & Frounfelker, 2019). Newly arrived non-French-speaking youths in Québec are received in welcome classes that provide a bridge for learning the language in a new culture, potentially creating a systemic gap with the regular program (Mamprin & Papazian-Zohrabian, 2024). This suggests the need to highlight their strengths, resilience, and creative potential rather than viewing these young people solely from the perspective of risk.

These adolescents are exposed to various adversities throughout their migration journey. The experience of space and time as inseparable aspects of being-in-the-world (Heidegger, 1927/2008) is constructed differently, marked by otherness and loss, for those who travel long roads. The World Health Organization [WHO] (2025) has highlighted the association of this population’s heightened vulnerability to mental health problems. According to Papazian-Zohrabian et al. (2018), well-being is not limited to happiness but encompasses adaptive abilities and relationship quality. These emphasize the importance of the dynamic link between well-being and mental health. Papazian-Zohrabian et al. (2024) suggested an ecosystemic approach to re-examine the socio-educational experiences of refugees and families seeking asylum, postulating that every individual possesses a genuine agency to act (Papalia & Martorell, 2018). Papazian-Zohrabian et al. (2018, 2024) highlight the value of the teachers’ actions and the school environment for the effective integration of refugee students. Through classroom observations and semi-structured interviews with teachers and newly arrived students, Koubeissy et al. (2025) addressed the importance of cultivating shared perspectives in research.

### 1.2. School as an In-Between Home

Schools play a key role in creating safe, supportive, and equitable learning environments, particularly for vulnerable students. Young people are receptive to the inquiry of existence; however, the current context is marked by geopolitical instability, armed conflicts, losses, and social crises that increasingly expose them to concrete and symbolic existential threats (Gomes et al., 2024). Thus, intercultural educational approaches are crucial to ensure an interdisciplinary perspective and emphasize sensitive pedagogy (Akkari & Radhouane, 2019).

In a qualitative study by Harpazi et al. (2020), 12 adolescents mentioned the following benefits of art therapy in a school setting: acceptance of art therapy as an outlet, personal and emotional engagement, calming and supportive alliance, haven of relaxation, and greater acceptance of the school experience. The authors reported that artistic materials and art-based experiences can help explore the implications of change. Beauregard et al. (2024) stressed the importance of offering newcomer children art therapy in a school setting to acquire agency. Riccardi and Gingras (2024) presented a case study in a school setting of a second-generation immigrant adolescent using the ETC framework, wherein choices of art materials and authenticity evoked losses and grief in a secure environment. Hence, the exploration of the phenomenological value of art process and product may give a new language to these adolescents (Gaete, 2022).

### 1.3. Creative Expression as a Momentary Home

Bachelard (1957/2001) revealed that the house as a symbolic space is our personal cosmos, reflecting our inner world and serving as a map of our hidden being—a theme echoed in art. Considering the properties of art materials when implementing art-based programs is fundamental because media are vehicles for creative expression and nonverbal communication (Lusebrink, 2010; Nan & Wong, 2021; Wong, 2021). Dion (2016) portrayed that a work of art offers an open perspective, a space for viewers to project their own experiences and emotions, as well as reflect on and examine their intimate lives. During this encounter, one develops the desire to welcome and receive others in their world (Jager, 1997).

In this regard, the ETC framework offers a creative space that integrates group member interactions with media choice, images, and art processes (Hinz, 2020; Riccardi & Rodrigue, 2023) This inclusive and culturally sensitive framework suggests that choosing artistic media, interacting with it, and producing images provides valuable insights into people’s thoughts, feelings, and actions across other spheres of their lives (Hinz et al., 2022; Whyte, 2020) enabling the retrieval and reworking of lived experience. Therefore, the way artworks are created, and their co-constructed meanings, becomes a mirror of the person’s worldview (Hinz, 2020).

The ETC adopts a person-centered therapeutic approach to develop a deeper understanding of one’s experiences; through this approach, the individual becomes an active agent in the healing process (Hinz, 2020). The framework highlights the therapeutic effects that manifest through visual expression (Kagin & Lusebrink, 1978; Lusebrink, 1990, 1991). This pan-theoretical model portrays individuals holistically, organizing their engagement with art materials and information processing. It suggests that using diverse materials and artmaking processes may evoke various sensorimotor, affective-perceptual, and cognitive-symbolic modes of interaction within a group setting (Hinz, 2020; Riccardi & Rodrigue, 2023).

In a literature review, Klauck (2021) underlined the ETC’s potential with refugees and asylum seekers. Through an ETC cultural lens, Phang (2025) reflected on her personal artmaking and Riccardi et al. (2025) addressed ruptures and repairs in therapy, acknowledging the importance of cultural humility.

**Figure 1:**
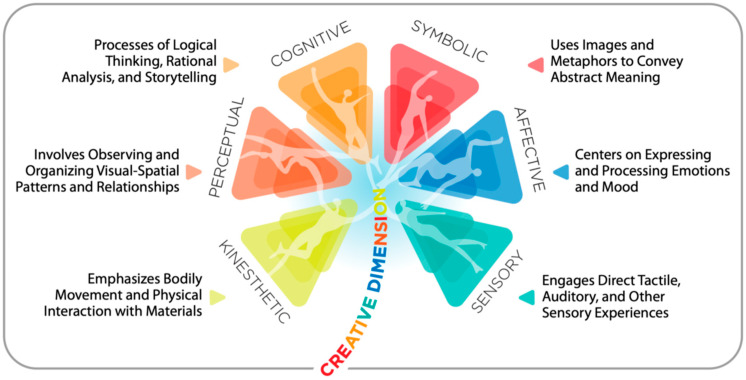
The Expressive Therapies Continuum (ETC) framework as inspired by Hinz (2020).

The framework is diagrammatically arranged along three levels (Kinesthetic-Sensory; Perceptual-Affective; and Cognitive-Symbolic) and one creative dimension of increasingly complex processing across these three levels, in the order listed (Figure 1).

Existing research helps us understand how the ETC can reduce psychophysiological stress (Nan et al., 2023) and negative emotional symptoms in different populations (Nan & Ho, 2017; Nan & Wong, 2021), increase psychological flexibility (Riccardi, 2013), and offer trauma care (George et al., 2025; Hébert, 2025). Phenomenological research on the ETC has also informed how participants perceive their lived experiences (Alfred, 2019; Grame, 2024). Emerging research on the ETC and cultural sensitivity emphasizes the importance of viewing therapist biases and self-knowledge within the framework and adopting a two-eyed approach (De Grand’Maison, 2020; Whyte, 2023). Ibrahim (2022) suggested the incorporation of the ETC as a theoretical and practical lens to plan art therapy interventions for refugee children and adolescents in a museum setting. Further, Hamouda et al. (2024) used a participatory action framework in temporary shelters for asylum-claimant children and teenagers. The results revealed that the choice of materials, as well as interactions, enable the participants to express their narratives and foster dialog in a relational space.

Our study builds on previous research by sharing the voices of the significant adults who shared the play space with 17 adolescents in a group-based program grounded in the ETC. In a school setting, the ETC framework can help conceptualize art-based workshops by categorizing interactions between the students, proposed media, produced images, and the art therapy process (Hinz, 2020). Furthermore, it links interactions with the school, family, and community, with school actors sharing the play space within the mesosystem (Bronfenbrenner, 1993; Mamprin & Papazian-Zohrabian, 2024). This framework enables a distinct understanding of newcomers’ history from a chronosystem perspective to address the temporal aspects of their development pre-, peri-, and post-migration (Papazian-Zohrabian et al., 2019). Through the lenses of a teacher, a special educator, and co-art therapists this research highlights the potential of the ETC framework to observe newcomer students’ interactions in an assortment of experiential and creative interventions based on media-dimensional variables of art materials and processes (VanMeter & Hinz, 2024).

### 1.4. Reflexivity as a Culturally Sensitive Posture

A qualitative research method allowed the researchers to address the lived experiences of the homeroom teacher, the special educator, and the art therapists regarding their participation in the program, and in relation to their perceptions of the young people’s engagement. Through active involvement in artmaking, having trained in cultural responsiveness and using response arts (Drapeau et al., 2020), a sensitive posture was taken by the co-art therapists in the face of the artistic productions made by the adolescents.

Additionally, the researchers’ and the art therapists’ logbooks allowed for continuous reflexivity to enhance the research process and maintain credibility, sincerity, and rigor (Fish, 2025; Tracy, 2010), and assist them in handling cultural alterity within the classroom. Caldairou-Bessette et al. (2017) highlighted that research in institutions with vulnerable populations, such as those in welcome classes, involves numerous ethical issues, such as data confidentiality, power relations between the researcher and participants, and a porous boundary between research and therapy. The logbook sheds new light on the interviews and supports the credibility of the study (Riccardi et al., 2025; Tracy, 2010). Engaging in self-reflective practice allowed the first researcher to recognize how their positionality shaped by migratory experience and theoretical framework generated implicit assumptions within the research (Bélanger-Dumontier, 2017).

## 2. Materials and Methods

### 2.1. Conceptual Framework

This phenomenology-informed qualitative study explored supportive adults’ perspectives on shared art-based workshops. Reflexive Thematic Analysis (RTA) was used to identify and interpret recurring thematic patterns in the data. More specifically, this study aimed to understand and describe the unique perception of the teacher, the special educator, and two art therapists regarding their experience of the ETC-guided workshops in relation to the young people’s experiences in the welcome classroom. A qualitative Big Q phenomenologically oriented approach guided data collection (Smith et al., 2022) and analysis, emphasizing the researchers’ active, interpretive role in identifying meaningful patterns across the data corpus (Braun & Clarke, 2021). The exploratory and inductive nature of the research allowed for the emergence of unanticipated themes (Bélanger-Dumontier, 2017). The individual’s lived experience is, at once, a co-construction from social interactions, and an utterly subjective process of construction of meaning from cultural values and imaginaries (Creswell & Poth, 2024). Finlay (2021) emphasized the researcher’s role as an interpretative artist. In a non-positivist qualitative approach, the researcher’s subjectivity is a resource for inquiry (Braun & Clarke, 2022). Titia Rizzi and Moro (2021) underlined that the act of drawing can be understood as both singular and universal forms of expression; therefore, the transcultural value of art therapy resides in the uniqueness that each artwork embodies.

### 2.2. Participants and Recruitment

Participants were recruited through a partnership with a multiethnic school in Québec, Canada, that offered more than 20 migrant welcome classrooms. The school coordinator facilitated initial contact with eligible classrooms. The inclusion criteria of the classroom were as follows: (1) composed of young people aged between 12 and 17 years old; (2) comprising students from newcomer families; (3) attainment of an appropriate level of fluency in French; and (4) full-class consent to participate in the arts workshops, including the teacher and the special educator. Considering the limitations of school-based research, a welcome classroom was chosen to identify a particular perspective (Smith et al., 2022). Hence, 17 adolescents newly arrived immigrants and refugees’ adolescents from 12 to 15 years old, originating from Haiti, Algeria, Mexico, Pakistan, Argentina, and the United States, participated in nine art-therapy workshops and a final exhibition, alongside several supportive adults. At the time of the workshops, they had been in the host country between five months and two years.

For this study, participants were the adults in the above students’ classroom. These comprised the homeroom teacher, who had more than 20 years of experience in welcome classes, and a special educator with more than 12 years of experience in working with immigrant teenagers, and 28 years in intervention. The participants also included two art therapists with 10 years of experience in community art-based art therapy. These adults were of Canadian origin and aged between 35 and 50 years old. With each adult, a semi-structured interview was conducted, from which the data were collected.

### 2.3. Procedure

This qualitative study aimed to describe, as an ecosystemic experience, art therapy workshops that used the ETC framework. This art-based research framework may take an ethical stance because it considers playing with diverse art materials as inseparable elements of dialog (Huard et al., 2024). The 17 participating adolescents co-created their migratory journeys through authentic choices between thematics and art. The study comprised two parts: art therapy workshops and nine semi-structured interviews with the adults who were actively engaged in the play. After the nine workshops, through semi-structured interviews, the teacher, the specialized educator and two art therapists shared their experiences of the workshops. The researcher’s observations deepened the understanding of the phenomena and established a secure base with the participants (Gagné-Trudel et al., 2024).

### 2.4. Art-Based Workshops

To approach the class in an authentic manner, the students, along with their teacher and special educator, participated in nine art therapy workshops (Table 1) led by two art therapists. The students were encouraged to draw on their cultural heritage at each level of the ETC, including choice of materials and theme construction. The art opening provided a playful, collaborative space to explore relationships with the self, others, and community. During the workshops supporting adults and art therapists acted as collaborators by also engaging with the students and the art materials. Through material choices and interactions, the adolescents shared their migratory journeys through the choice of materials and themes, they shared their relationships, school social network, and reflections within relational and family circles.

### 2.5. Interview Guide

In-depth semi-structured interviews lasting 45 to 60 min were embedded with cultural awareness (Whyte, 2020) and conducted to explore participants’ experiences (Smith et al., 2022; Table 2). These interviews were recorded, transcribed verbatim, and anonymized; the findings provided combined perspectives of the four adults while drawing on the students’ experiences and their perceptions of the students’ experiences. From this perspective, the meaning was co-constructed to conform to general themes through active interpretive engagement with the narratives, while retaining the researcher’s voice.

### 2.6. Data Collection

During data collection, the first author remained sensitive and flexible, fostered dialog, and facilitated co-construction (Smith et al., 2022). By exploring the experiences from the benevolent gaze of those who attended the workshops, a detailed and multidimensional account of the students’ experiences along their own reflections were also unveiled (Smith et al., 2022). The reflexive journal, a collection of artworks, and semi-structured interviews comprised the collected data.

### 2.7. Analysis

Guided by Braun and Clarke’s (2021, 2022) six-phase approach, the study employed RTA, which was chosen for its epistemological flexibility (Braun & Clarke, 2006, 2021). Within a larger methodology based on a phenomenological lens, it allowed for the co-construction of a more nuanced narrative of art workshops from the perspective of the supporting adults. Therefore, RTA facilitated the researcher’s perceptions of the interplay between the participants’ narratives, research question evoking the encounter, and coping strategies used in the workshops.

All interviews were audio-recorded and later transcribed manually by a trained researcher. Transcripts were reviewed by a second researcher alongside the original audio recordings to ensure accuracy. All spoken content as well as additional linguistic features such as pauses, laughter, and hesitations were included within transcripts. These elements were removed at the manuscript writing stage to enhance clarity and readability within excerpts, without changing the wording or meaning of participants’ responses.

Qualitative data analysis was conducted using NVivo 15. A trained researcher completed the primary coding of all transcripts, using inductive coding process. The researcher then conducted secondary and tertiary coding to organize codes into broader themes. After each stage of the coding process, a second researcher reviewed the codes and provided feedback to ensure consistency and adequate structure. Final coding decisions were determined collaboratively through discussion until consensus was reached. To protect participant confidentiality, all identifying information, including names, were removed during the transcription process. Audio files, transcripts, and NVivo project files were password-protected and were only accessible to members of the research team.

Additionally, the 16-item checklist proposed by Ahmed et al. (2025) served as a practical tool to ensure adherence to the established thematic analysis steps. This study followed the COREQ guidelines for reporting qualitative research (Tong et al., 2007; Von Elm et al., 2007). Participants validated the findings. Table 3 shows the theme development and interpretation, and the researcher’s reflexive posture.

## 3. Results

The RTA revealed three central themes describing the adults’ perspectives in their relationships with the students and the art-based experience: (1) an emancipatory experience, (2) a transitional play experience, and (3) an intersubjective experience (Table 4).

### 3.1. Theme One: Emancipatory Experience

The expressive accounts of the school actors highlighted that the workshops enabled adolescents to have an emancipatory experience, encouraging them to embrace their true selves. The first subtheme reflected the interplay between artistic expression and relational presence; the second demonstrated the adolescents’ shift from imposed limits to self-directed forms of action and meaning. This fostered a holistic experience via body-mind-spirit (Kagin & Lusebrink, 1978). In total 136 individual images were created, four in subgroups and one as the full group.

#### 3.1.1. Subtheme One: The Practice of Freedom

The workshops supported the students in developing autonomy within their supportive relationships. Artmaking as a freeing and liberating practice was the most prevalent theme across all transcripts. It involves the idea that a non-directive approach is key to allowing students to let go and act autonomously in ways that they may not have been able to in the past. The adolescents became aware of the systems that shaped them and started making conscious choices. This theme, underlying safety, self, and co-regulation, was illustrated by an art therapist:

“They always said yes to what we wanted and then decided that they were going to say no that day. We did not fully understand this phenomenon. We asked the question again and re-explained to see if it was because they did not understand what we were trying to tell them. However, they did not intend to play a prank on us. […] The point of making a choice, to assert, ‘I like…’ Maybe it’s just to be heard and to have a voice. It’s probably not them who made the decision to come here; it’s not them who made all the decisions that influenced their lives. There’s surely a lack in that regard, for them to have the ability to choose.”

**Figure 2:**
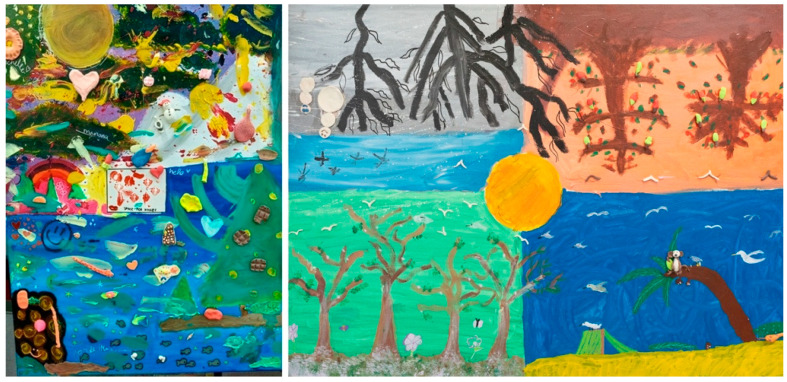
Subgroup projects, mixed materials, 22 × 28 inches.

The supporting adults observed a progressive posture of comfort over time and in the liminal space as the students expressed themselves through their created images. Looking at her logbook, the workshops also brought difficult emotions to the surface for this art therapist: “I wrote: “protect the naivety of childhood, this fragile little creature.” The freedom offered made her wonder, “Am I doing enough?” “Do they need more than I can give them?” The co-art therapist “noticed across the workshops that by using the art materials, they became more comfortable; maybe with us as well.” The homeroom teacher said that the workshops became spaces for agency (see Figure 2):

“In the workshops, they said, ‘I exist. I am also able to do things.’ I tell myself, they changed country, you know, there is a lot of pressure from their parents, ‘You must do well. We came here for you.’ But it was like ‘We are giving you all your space’.”

Embracing this freedom, the students began building and creating their desires. The supporting adults stated that they were able to express themselves through the artmaking process. The co-art therapist also portrayed their need to fill the page, as well as their fears related to slowing their pace.

“… I noticed the thirst to produce… there were some who were really focused… and [concentrating] much more in production, not necessarily in terms of quantity, but the layers. There are some who produced a lot, and quickly. There was perhaps something for some [in terms of a difficulty in tolerating, either absence, or emptiness…I’m talking about the difficulty in tolerating the blank page.”

The specialized educator expressed that the adolescents “may not be used to freedom.” Thus, “being able to find something that they loved within the art materials” was a way to reimagine oneself beyond imposed narratives “and gave them the chance to express themselves.” The homeroom teacher explained:

“For some, it was clay. For the others, it was a painting. Oh my God! They really let themselves go, and we had to stop them. So, I saw many little humans trying many things and, in the end, they were able to say: ‘Well, for me, this is what I prefer.’ I really liked that in the end.”

#### 3.1.2. Subtheme Two: The Art of Choosing

As the creative process evolved, another layer of meaning emerged through the dialog around the choice of art materials. The selection of materials and themes conveyed the idea that a non-directive approach is key to letting go and acting autonomously in ways that these adolescents could not do before, allowing them to lessen their apprehensions. The specialized educator captured this premise: “It’s just about supporting them in their creations. I think what helped them the most was that they had a choice.” The homeroom teacher corroborated this experience: “what I enjoyed the most was introducing them to different materials … letting them touch everything and then seeing what they liked.”

Altogether, the participants noted that the art experience allowed the adolescents to freely express themselves through their art, without any school demands. One art therapist recalled, “We made several efforts to talk about what a mistake is, and that there isn’t such a thing as a mistake.” The co-art therapist recognized that choosing a particular medium served as an avenue for self-expression:

“I got the impression that we offered a space in which we invited them to participate freely. […]. Here are different mediums you can experiment with, so have fun and just express yourself in whatever way you want. In my opinion, this was beneficial.”

A central thread in the transcripts highlighted that artmaking allows students to act freely without any apprehension toward consequence or guidance and artistically create on their own terms. The special educator observed, “At the beginning, they were trying to get validation from their teacher, and afterward they immersed themselves completely.” This was echoed by the homeroom teacher: “That’s when they really let go [with watercolor painting]. ‘Okay, I have the right, it’s allowed.’ There are no consequences. We don’t say, ‘you can’t do that; you can’t do it…’ No, ‘go for it!’”

The workshops provided a safe space for the students to reclaim their voices, authorship, and presence. “It’s the aspect of leaving them without rules, a little freer,” revealed the specialized educator. One art therapist underlined that “the moments when they really expressed their choices … when they took an initiative or decided something for themselves, those were the moments, for me, were the most touching.”

### 3.2. Theme Two: Transitional Play Experience

Overall, the art-based workshops offered art materials and experiences that transcended events and time barriers that might have hindered communication and expression. All adults observed that creating together enabled the adolescents to gain insight into their inner world and noted their own felt experience. The first subtheme considers media interaction and image formation, giving the adolescents a voice in identifying their unique experiences across the landscape. The second subtheme denotes a temporal playground where they could reclaim memories left behind.

#### 3.2.1. Subtheme One: Navigating Barriers Through Expression

The interviewees mentioned that the students expressed themselves through the artmaking process. The educator recounted that in the reception classroom, “there were times when the language of communication was difficult. The arts really act to help students express themselves.” Echoing this reflection, the homeroom teacher shared that the students rarely talked about their experiences in the day-to-day classrooms; however, in the workshops, their images acted as their voices [with watercolor painting]:

“It’s rare that they’ll talk about it. […] Some have made this huge journey on foot. They experienced adversity and kept a certain shell. So, I tell myself that across their drawings and paintings, they can express something, and it is beneficial for them.” One art therapist also expressed; “Hearing those little bits of horror here and there, and then thinking, oh my god, why am I here, why was I born here? And then seeing them navigate adolescence through that.”

**Figure 3:**
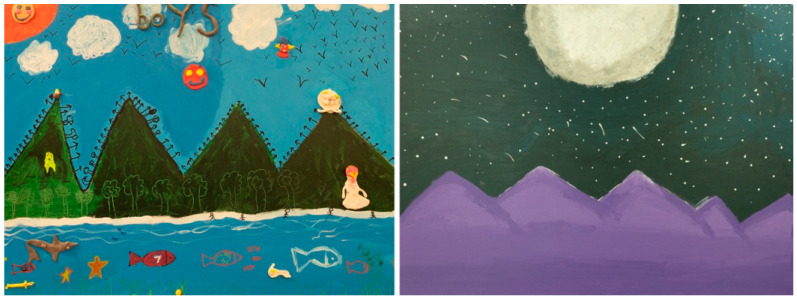
Subgroup projects, mixed materials, 22 × 28 inches.

The dialog deepened when the art therapists underlined the recurring reminiscent symbols in the artworks. One art therapist mentioned that the workshop “was like starting an adventure, taking a journey, braving the storm. It’s so touching to see them try, make mistakes, and learn from these obstacles.” Similarly, the co-art therapists mentioned that the students shared visions of the landscapes they encountered (see Figure 3):

“We even saw it in the mountains; one started with a symbol, and it came up in the others’ artworks. Thus, there is a message that goes beyond language to form a bond between them through the art materials. It was a beautiful experience seeing them work together and discover themselves through imagery. To see them take risks and try new things.”

Art materials and image-making helped the students represent their uniqueness and relatedness. The homeroom teacher “discovered them through the art materials.” They noticed that “language is not the only way to express oneself.” When creating individual aprons,” some really put a lot of things that were more personal…others asked for everyone to hallmark their handprint … some wanted the entire classroom on their apron.” In contrast, the co-art therapist expressed that working in a group may also have retained some barriers that were slightly visible:

“Then, I think that’s when their defenses kicked in, or they became like, in that attitude of: Oh, but we’re so close to touching something,” and then, whoosh, you know, the barriers come down. At the same time, I understood, because we were in a classroom, there are other worlds, and it’s not a context where they want to be vulnerable.”

Expanding on the students’ distinctiveness and usual aversion to not being seen, the educator mentioned that the “free environment deserved to be exploited further … [they] had the impression that they discovered themselves … no matter what anyone else said or thought about it.” This resonated with the art therapist’s interpretation that “it was necessary to propose different materials […]. These acted like symbols or metaphors for all the identities and in different ways to express oneself.” The homeroom teachers developed the idea of uniqueness and bravery:

“There was no pressure from this workshop, and nobody said, ‘Oh no, I’m not capable.’ When we work on the French language, they say, ‘I’m not able to, it’s too hard.’ But here … everyone let themselves go, and everyone played the game. […] So, they [created] some jewelry [with clay], put on nail polish with the paint, [created] fake tattoos on their arms … ‘We all looked the same along with 1800 students in the school, but now, I have the chance to show [who I am]’.”

#### 3.2.2. Subtheme Two: Reclaiming Oneself Through Play

Art was a means of expressing oneself and revealing one’s uniqueness. It was used to express life in one’s country of origin. An art therapist mentioned that “a space like this is necessary, and it doesn’t exist everywhere. These workshops created this space.” She also mentioned that “sharing the space with a colleague is a privilege and, being able to handle unforeseen circumstances means that no one is penalized.” The teacher explained how art was used to express the adolescent’s origins:

“This was an occasion for them to think about their country and represent it. There are several students who didn’t come to school right away … Across nature, a bit of their country was hidden beneath it … whether it’s in the narratives or drawings, their country always comes back. For me, it’s a beach with palm trees; [for them] it was their country, and it was the beach where they would go with their families. Digging a little deeper, this sense of belonging to their country was apparent in the works they created.”

One art therapist unearthed the following through the adolescents’ gestures and imagery: “Sometimes it was in the artwork … for others it was just from their silence, in the way they carried themselves.” The co-art therapist added, “It was necessary to propose different materials, different ways of expressing oneself.” The specialized educator expressed, “[these adolescents] found themselves through movement and play.”

Through the art-based process, the adolescents also confronted the delays they face in entering school. Hardship and pain were part of their lives, which they expressed in their images. Their stories appealed to the temporality linking human experiences, the ways in which they told their stories, and the affective dimensions related to these experiences.

### 3.3. Theme Three: Intersubjective Experience

The supporting adult experienced that the students gradually revealed themselves as artists and became more receptive to working with others. The first subtheme posited that art-based experiences allowed them to change the relationships they fostered with their own stories and transition to a class dynamic. The second subtheme indicated that art-based workshops provided a liminal space for building a sense of community.

#### 3.3.1. Subtheme One: Being-with-Others

Interactions among classmates and connections with significant adults supported the young individuals in nurturing relationships and developing a sense of trust in the art process. The teacher felt: “as a mother holding the bicycle, and then at some point, letting go, and then they must keep pedaling.” She underlined that: “I have a very good connection with my students […] a little family atmosphere. […] I really enjoyed working (making art) with them, I made sure to move around, to see if everyone was able to find their place”. The specialized educator explained that this project “was created within a community that brought them together, even if for some, [artmaking] wasn’t the biggest strength, they did it with purpose.” One art therapist celebrated the adolescents’ audacity, mentioning that they were “very courageous, because all of them enrolled.” They added that they were “very generous to participate in the workshops.” The co-art therapists shared similar perspectives:

“You can’t help but notice their generosity and courage. The students didn’t know what to expect in the presence of their classmates or therapists, who did not know them very well, to ask them to jump right in the splash, in the unknown … creating their signatures. Creating artwork is a beautiful symbol of courage. It fosters their creativity and ability to express themselves.”

A sense of trust developed as the workshops unfolded; the specialized educator noticed that “at the beginning, they were a little stuck and didn’t know much. It’s like you all showed trust in them: ‘Ah yes, go for it.” An art therapist mentioned, “as the game progressed, this bond of trust was established between us, and between them.” The transcripts were rooted in the adolescents’ migratory journey and suffering, and in their desire to rediscover themselves through a horizontal practice of care with others, despite the turmoil of living far from their country of origin.

#### 3.3.2. Subtheme Two: Sharing Space, Finding Place

All the participants expressed that sharing art materials and imagery provided opportunities for support and encouragement. The students became more comfortable with art materials when working with other students and adults, thus developing a deeper sense of community. They were guarded at first; however, as the workshop progressed, they adopted a more comfortable and grounded posture. This is illustrated in the following excerpt from the specialized educator: “I saw the progression and the group dynamic change across the different workshops and mediums. I had the impression that I was a spectator [in a play].” The homeroom teacher explored her experience of taking an active posture:

“To participate with them, I really enjoyed it. I think they liked it too “ Okay, the teacher is on board, that’s cool.’ I think it’s important in a project like this, not to… just sit in the corner of the classroom and be a spectator.”

Teamwork and comfort were developed throughout the workshops. An art therapist noted, “There was this sense of collaboration and teamwork toward a common goal. Group identity emerged through artmaking.” The co-therapist noticed that the workshops provided, “a human-to-human sense of community. It’s like building a little community, and [all the adults] tried to be a part of that community.” The homeroom teacher noted:

“Everyone is helping each other; everyone is in the same boat. I find that beautiful. They help and encourage each other. Even during the project [their voice was heard] ‘No, it’s okay, you know, restart. No, it’s nice.’ It’s really like a small family. This created some beautiful exchanges.”

**Figure 4:**
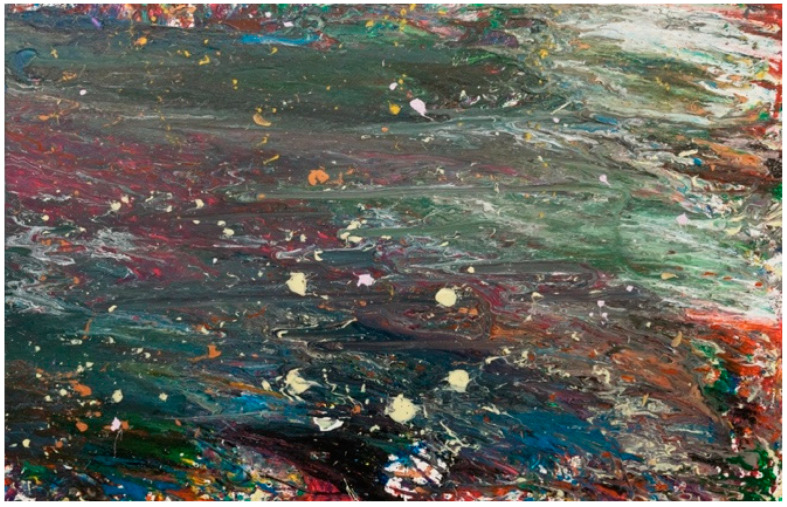
Group project, mixed materials, 30 × 36 inches.

Expressive undertakings provided opportunities for festivities in the classroom and in the art exhibition, where perceptions, thoughts, and feelings were transformed when the adolescents and the adults entered an intersubjective world (see Figure 4). The art therapist explained how group alliances were formed:

“[At the beginning of the workshops] they were often seated on their desks. They were doing their things. [Later in the workshops], they were all standing up and making jokes. They were making [and wearing] clay hats. And suddenly [in the opening], they were all standing up and walking around to interact with each other.”

This theme revealed that the co-construction of stories in a relationship of alterity gave meaning to a hybrid tale arising from the personal migratory journey at the crossroads of others’ personal tales. In the educational context, the art workshops became places for existential conversations between the students and their proximal community. The shared experience showed that the supporting adults openly expressed their experiences of the workshops, noting how the students themselves became the central phenomenon. Their reflections highlighted how the play with materials as a form of performance captured their attention and shaped the overall sense of community. Through their own interpretations, the supporting adults revealed not only what they experienced but also how the students influenced their understanding and engagement with the image-making process.

## 4. Discussion

This qualitative study identified that the workshops provided a liberating emancipatory experience, a transitional play space to overcome language barriers, and the emergence of an intersubjective experience.

These themes highlight that the ETC framework provides a collaborative and respectful therapeutic process that fosters ongoing self-reflection (Hinz, 2020). Adults perceive the classroom as a playground that encouraged self-reflexivity and honored multiple ways of knowing through choices and indirect guidance (Whyte, 2020). Using a culturally safe approach ensures that diverse communities feel that they have choices, voice, and empowerment (Stepney, 2022). Ultimately, through relationships with the self, others, and their proximal community, the displaced population can establish an engaging and intersubjective world (Jager, 1997). These experiences appear in various forms across the interviews, suggesting that this is a widespread phenomenon. The adults’ perceptions also suggest that the adolescents’ ability to act and choose freely in the workshops helped them express painful emotions and provided them with the freedom to author their own lives (Grame, 2024; Hamouda et al., 2024; Yalom, 1980).

The first theme depicted that the practice of freedom and art of choosing enabled by the supporting adults offered the adolescents a liminal space for creation, gestures, and material engagement through sensorimotor and affective encounters (Hinz et al., 2022). Conversely, as the art therapist emphasized, taking a developmentally supportive approach rooted in presence and granting young people greater freedom can at times feel uncomfortable for the therapist. According to Gadamer (1960/1996), a game’s purpose is fulfilled when the player forgets themselves in the play, with the art being part of a historical and spatiotemporal extension. Dion (2016) provided an analogy between psychotherapy and the creative process: a discovery made through play in a hospitable setting that allowed creativity and transformation. Therefore, all experiences are embodied, situated, and caught in a relationship of intentionality with the world (Merleau-Ponty, 1945/2012). Consequently, experiencing the world in and through a suffered body colors its entire relationship to the world (Vinit & Quintin, 2016). The ETC framework may have provided a context to understand emancipation as the lived experience of the adolescents within social and bodily experiences. Accordingly, Haeyen (2024) stressed the role of co- and self-regulation through creative art in understanding safety, play, and regulation through embodied processes. Porges (2022) stated that, based on the polyvagal theory, a safe environment associated with positive social interactions can optimize health, growth, and restoration. Consequently, the adults emphasized that the adolescents felt safe in co-constructing their world through body- and affect-based experiences.

The second theme indicated that the ETC framework provides an understanding of meaning through play, in which the adolescents could navigate language and cultural barriers. They recreated their stories through artmaking experiences. These findings corroborate Grame’s (2024) qualitative study that art elements and materials were the primary means of communication for adolescents with trauma. From the emancipatory space to existing on their own terms, they allowed themselves, through play, to cross the language barriers that had previously limited them. By co-creating alongside students, the supporting adults discovered that a safe and playful environment emerged—one in which adolescents felt free to express themselves even when facing adversity (Beauregard et al., 2024). It reminds us that these adolescents understood the world based on historicity; thus, their understanding of the present moment emerged from their journey. Marin and Zaccaï-Reyners (2013) emphasized that the ability to assign meaning to our existence despite the pain makes suffering meaningful. Isolated from the worlds they knew, the adolescents described their feelings through the portrayed images, accepting the possibility of taking risks and making mistakes (Bertolini, 1996). Therefore, through playing together as a community with the art materials, there was a possibility of creating a new feeling of belonging. The process of adapting to the external world by creating a sequence of physical and imaginal spaces helped the students symbolically exit the classroom. These affective scenes depicted places they had left behind, newly encountered, and envisioned anew. Together, with the significant adults, their works convey a layered story of adaptation, creativity, and reminiscence.

The third theme is aligned with finding the essence of fostering community support and being-with-others. Through their reflections, the adults recognized that the adolescents found a place to exist through communal creation. An informed framework within an ecosystemic approach can describe the complex and multifaceted types of traumas as a direct influence of societal oppression and its impact on the entire macrosystem (Kalaf & Plante, 2019). It links micro-, meso-, and eco-systems by highlighting the social and cultural values that underpin the development of a person (Bronfenbrenner, 1993). In this context, the chronosystem acknowledges the entire migratory process, providing a holistic lens system (Bronfenbrenner, 1995). In the school system, the workshops were based on a horizontal practice of collaboration, where the students, teacher, educator, co-art therapists, and families became participatory actors at the final art exhibition. Through the voices of nine teachers, Audet et al. (2024) highlighted the importance of respect, trust, valuing students, drawing on students’ first languages, giving them a voice, and working with families in schools. In this transtheoretical model, the relationship between temporality and history is fundamental. Therefore, the ETC can serve as a guiding and safer framework for exploring the complex trauma that allows immigrants, asylum claimants, and refugees to rewrite and reappropriate events in their lives in a community setting (Klauck, 2021). These results are consistent with Kassan et al. (2023)’s phenomenological study findings about the lived experience of newcomer youths who graduated from high school, revealing the interconnectedness of school actors, peers, community, and family.

The analysis of these three themes demonstrated that art workshops provide a transitional environment to reconstruct imaginary landscapes. Consequently, art materials function as a shared medium through which multiple languages intersect, fostering connections between the self and others. Instead of using art materials to respond to a linear timeline change, the young adolescents used these to create a lived space for self-reflection, discovering that their path was far from linear. The significant adults shared that these adolescents showed bravery in the presence of others, crossing barriers composed of phenomenological suffering interwoven with stories of loss and grief. Through the workshops and the art materials, the adolescents were able to experiment with intersubjective experiences. Within accompaniment, artmaking offered alternatives, but also the ability to choose the action of not choosing, to withhold action or to choose silence. This emancipated world was achieved through choices and creative autonomy to facilitate reminiscence. Thus, the ETC framework guided by two art therapists, a teacher, and an educator enabled the exploration of students’ diverse backgrounds and supported the reconstruction of their meanings through agency.

### 4.1. Strengths, Limitations, and Recommendations

This study used in-depth, semi-structured interviews within a constructivist-interpretative paradigm. Although it allowed for the co-construction of the adults’ experiences of art workshops with the first researcher’s interpretations, only a single interview was conducted. Therefore, it would be hasty to consider the long-term repercussions of the workshops. It is important to address the assumptions of the first researcher when they were exploring the transcripts with their own apprehensions. Reflexivity was oriented toward self-awareness of biases, research participants’ dynamics, coding transparency, and influence of the sociopolitical context (Olmos-Vega et al., 2023). Maintaining a self-reflexive journal and collaboratively examining coding decisions and assumptions between the authors. As an illustration, during the initial phase of coding, the research team identified navigating barriers through expression and reclaiming oneself through play as potential themes. One researcher suggested that these processes may be closely aligned. Therefore, the research team reached a consensus and determined that they should be categorized under the broader theme of playful experience.

An important exploration of disconfirming evidence in the transcripts helped recognize that the present school environment was part of the workshops. The homeroom teacher mentioned that during integration into mainstream education, “a certain routine conveyed a sense of security … the workshop came at the perfect time. That day, the colors were much more somber, and we used the paintbrush. I think that their emotions were really reflected onto their artwork.” The teacher also shared an experience of the school community: “I think that it was good that the workshop was held of the day after the violent event (a student was seriously injured in an attack after school), it helped change people’s thoughts.” Consequently, cultural differences, power, privilege, and unresolved tensions intersected in the art therapy workshops (Kapitan, 2022). Furthermore, the interview process may have influenced the analysis owing to the power dynamics between the researcher and participants. For future research, the art therapist suggested offering the workshops to the school actors first then the students to “experience firsthand the workshops, because that is always the big question, the difference between an art therapy and an art class.”

### 4.2. Conclusions

The objective of the study was to explore the experiences and perceptions of a homeroom teacher, a special educator, and two art therapists who participated in art-based workshops in a welcome classroom for newly arrived adolescents, and to understand the significance these workshops held for them as well as for the adolescents they serve. This qualitative study gave voice to the supporting adults who shared that the workshops offered a culturally safe, embodied, and relational art therapy framework that supported newcomer adolescents in navigating trauma, language barriers, and reconfiguring one’s sense of self within a school context. Through their active participation in workshops designed to offer play and opportunities for self-discovery, the supportive adults developed a deeper awareness of the need to allow adolescents to express their autonomy, an understanding that emerged from their lived experience within the process.

Through artistic freedom and choice, adolescents accessed a liminal and playful space that fostered self-regulation, emotional expression, and meaning-making beyond linear narratives of adaptation. Art materials functioned as a shared, translinguistic medium that enabled storytelling, reminiscence, and risk-taking, while communal creation nurtured intersubjective connections and a sense of belonging within broader ecological systems. The results suggested that the ETC framework facilitates emancipation not as an abstract concept but as a lived, relational, and embodied process, allowing adolescents to author their own lives through creative autonomy and community engagement. The study emphasizes the significance of constructing safe spaces in school settings where students and their supportive adults can play together, creating ongoing collaborations within the co-creation of a horizontal practice of care. Despite methodological limitations, this study underscores the potential of ETC-informed art workshops as a respectful and collaborative practice for supporting immigrant and displaced youth, and it calls for further longitudinal research to explore their sustained impact within educational and community settings.

## Figures and Tables

**Table 1:** Art therapy workshops.

Workshop Date	Collaborative Interventions	Proposed Art Materials
13 December 2022	Individual drawings	Pencils and acrylic markers
18 January 2023	Individual personalized aprons	Acrylic markers and paint
24 January 2023	Individual personalized aprons	Acrylic markers and paint
	Individual emotional immersion	Watercolor paint
30 January 2023	Individual emotional immersion	Watercolor paint
	Painting in subgroups	Acrylic markers, paint, sculpting materials
6 February 2023	Painting in subgroups	Acrylic markers, paint
17 February 2023	Modeling in subgroups	Light modeling clay
	Artistic discovery	Choices *
20 February 2023	Individual and subgroup finishing workshop	Choices *
22 March 2023	Artistic discovery	Choices *
21 May 2023	Closing workshop in the class group	Acrylic pouring and natural elements
21 June 2023	Community opening	Expressive arts (artmaking and dance)

* Choices: lead and colored pencils, dry pastels, watercolor paint and pencils, acrylic paint and markers, images, and light air-drying modeling clay.

**Table 2:** Outline of the interview.

**Introductory sentence:** This study aims to better understand the experiences of newcomer students in high schools through art-based workshops. Can you share your experience of the workshops and the presence of arts for newcomer youth?
**Open questions on the workshop experience:** Can you tell me about your experience with the workshops? How did these workshops allow students to share their stories? What were the main challenges faced during the workshops? In addition to your experience with the art-based workshops, we would like to learn more about the implications of art materials and processes.

**Table 3:** Theme development, interpretation, and the researcher’s reflexive posture.

Phases	Analysis (Braun & Clarke, 2021)	Reflexivity (Tracy, 2010)
Familiarization	The first author transcribed and recurrently reviewed the data corpus on a smaller sample of transcripts.	Observations were recorded in a reflexive journal.
Initial code generation	The inductive-driven analysis included dividing the data into units that were systematically and iteratively identified, creating initial codes.	A dialog with the research laboratory team encouraged reflexivity. Codes were developed in NVivo15.
Initial theme construction	The search for connections, alliances, and broader patterns of meanings was developed into initial themes capturing a wide range of narratives.	Art-based reflections (Fish, 2025) and journaling encouraged self-reflexivity to address biases.
Initial Reviewing and refining the themes	Themes were redefined and reviewed. Therefore, a nuance was developed to answer the research question, aiming for conceptual depth.	Focusing on the research question enhanced the rigor of the study. After each stage of the coding process, a second researcher reviewed the codes and provided feedback.
Defining and naming the themes	This phase involved theme development, comprising refining, defining, and labeling. An abstract was developed for each theme.	Themes were developed for an aesthetic representation of the data corpus to address resonance.
Producing a narrative report	Through a meaning-based interpretive story theme, the analytical narratives were woven with the literature to present a nuanced understanding of the art-based workshops.	In-depth illustrations of the transcripts addressed the credibility of the research in connection with the literature for meaningful coherence.

**Table 4:** Themes and subthemes revealed in the thematic analysis.

Themes	Subthemes
1. Emancipatory experience	1.a The practice of freedom 1.b The art of choosing
2. Playful experience	2.a Navigating barriers through expression 2.b Reclaiming oneself through play
3. Intersubjective experience	3.a Being-with-others 3.b Sharing space, finding place

## Data Availability

The interview data on which the study is based are not publicly available due to privacy regulations. The data presented in this study are available on request from the corresponding author due to ethical considerations regarding the protection and privacy of a vulnerable participant population.
